# Nature’s Bioactives in Cardiorenal Syndrome: Polyphenols at the Crossroads—Preclinical Insights into Redox, Inflammation, and Mitochondrial Protection

**DOI:** 10.3390/nu18060955

**Published:** 2026-03-18

**Authors:** Caterina Carollo, Maria Elena Ciuppa, Alessandra Sorce, Salvatore Evola, Emanuele Cirafici, Maria Giovanna Vario, Roberta Scimeca, Rosalia Lo Presti, Giuseppe Mulè, Gregorio Caimi

**Affiliations:** 1 Department of Health Promotion, Mother and Child Health, Internal and Specialistic Medicine, Unit of Nephrology and Dialysis, Policlinico “P. Giaccone”, University of Palermo, 90100 Palermo, Italy; mariaelena.ciuppa@community.unipa.it (M.E.C.); alessandra.sorce@community.unipa.it (A.S.); emanuele.cirafici@community.unipa.it (E.C.); mariagiovanna.vario@policlinico.pa.it (M.G.V.); roberta.scimeca@community.unipa.it (R.S.); giuseppe.mule@unipa.it (G.M.);; 2 Catheterization Laboratory, Department of Medicine and Cardiology, Azienda Ospedaliera Universitaria Policlinico “P. Giaccone”, 90127 Palermo, Italy; salvatore.evola@policlinico.pa.it; 3 Department of Psychology, Educational Science and Human Movement, University of Palermo, 90100 Palermo, Italy

**Keywords:** cardiorenal syndrome, polyphenols, Nrf2, NLRP3 inflammasome, mitophagy, oxidative stress

## Abstract

Background: Cardiorenal syndrome (CRS) represents a complex clinical entity characterized by the bidirectional dysfunction of the heart and kidneys. Despite advances in pharmacological therapy, CRS remains associated with high morbidity and mortality. Pathophysiological drivers, including oxidative stress, chronic inflammation, and mitochondrial derangements, create a self-perpetuating cycle of organ damage that necessitates multitarget therapeutic approaches. Objective: This review synthesizes current preclinical evidence regarding the protective roles of plant-derived polyphenols—specifically bergamot, curcumin, quercetin, catechins, and resveratrol—in mitigating the cardiorenal continuum. Methods: An analysis of recent literature was conducted, focusing on the molecular mechanisms by which these bioactives modulate redox balance, inflammatory signaling, and mitochondrial homeostasis in experimental models of CRS. Results: Polyphenols act at the crossroads of several stress-response pathways. Key mechanisms include the activation of the Nrf2/HO-1 axis to enhance endogenous antioxidant defenses, the suppression of the NLRP3 inflammasome to attenuate systemic “inflammaging”, and the preservation of mitochondrial quality through SIRT1/PINK1/Parkin-mediated mitophagy. Furthermore, emerging evidence highlights the role of polyphenols in modulating the gut-kidney-heart axis by reducing microbiota-derived uremic toxins. Conclusions: Preclinical data suggest that polyphenols are potent multifunctional agents capable of breaking the feedback loops of cardiorenal injury. While bioavailability remains a significant translational challenge, novel nano-delivery systems and synthetic analogs offer promising strategies for clinical application. Integrating these bioactives into CRS management could provide a decisive adjunctive strategy to improve metabolic homeostasis and prevent end-stage organ failure.

## 1. Introduction

**Figure 1:**
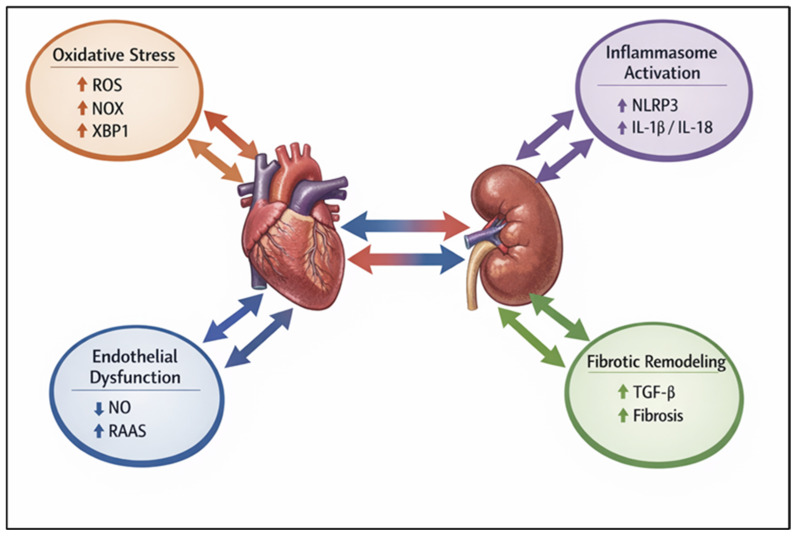
Pathophysiological network of cardiorenal syndrome. The heart and kidney are interconnected through a complex bidirectional crosstalk involving oxidative stress (ROS, NOX, XBP1), inflammasome activation (NLRP3, IL-1β, IL-18), endothelial dysfunction (NO, RAAS), and fibrotic remodeling (TGF-β, extracellular matrix deposition). Colored nodes highlight key molecular pathways, while arrows indicate their simultaneous impact on both organs, providing a mechanistic overview of CRS progression.

Cardiorenal syndromes (CRS) describe the complex bidirectional interaction between the heart and the kidneys, whereby acute or chronic dysfunction of one organ induces structural and functional impairment of the other [1]. Although the close relationship between cardiac and renal function has been recognized for decades, the formal classification of CRS was introduced in the early 2000s to better characterize the heterogeneous phenotypes of this condition [2,3,4]. Systemic disorders such as diabetes mellitus, hypertension, and atherosclerosis frequently act as common drivers of concurrent cardiac and renal injury, highlighting the symbiotic pathophysiological relationship between these organs [5]. Epidemiological studies indicate that CRS affects a substantial proportion of patients, with an estimated prevalence of up to 0.4% in the general population and 2–3% in individuals with diabetes and heart failure, and is associated with increased morbidity and mortality [6,7]. CRS are classified into five types: acute cardiorenal (type I), chronic cardiorenal (type II), acute renocardiac (type III), chronic renocardiac (type IV), and secondary systemic CRS (type V) [1,2]. These phenotypes are driven by distinct dominant molecular mechanisms: in Type II CRS, chronic cardiac dysfunction promotes renal injury primarily through persistent venous congestion, neurohormonal overactivation, and sustained hypoxia, which together trigger local endothelial dysfunction and fibrotic signaling; conversely, in Type IV CRS, primary chronic kidney disease drives cardiac impairment predominantly via the accumulation of uremic toxins, profound systemic oxidative stress, and mineral dysregulation, which collectively accelerate vascular calcification and left ventricular hypertrophy [1,2]. Most preclinical studies investigating polyphenolic interventions have focused on type IV and type V CRS, particularly diabetes-induced chronic kidney disease with associated cardiovascular dysfunction [8,9]. To elucidate the pathophysiological sequence driving CRS, the underlying molecular mechanisms can be stratified into a conceptual hierarchy, progressing from upstream systemic triggers to downstream structural effectors. The initial tier comprises systemic and hemodynamic perturbations—such as neurohormonal overactivation, persistent venous congestion, and the accumulation of uremic or metabolic toxins—which serve as the primary extracellular instigators of cellular injury. These systemic insults subsequently precipitate a secondary tier of intracellular stress responses, fundamentally characterized by organelle dysfunction [10]. Specifically, mitochondrial impairment compromises cellular bioenergetics, alters mitophagy, and disrupts metabolic pathways, including AMP-activated protein kinase (AMPK) and sirtuins (SIRT1/3) [11,12,13], while endoplasmic reticulum stress triggers the unfolded protein response via the transcription factor X-box binding protein 1 (XBP1) [14]. This organelle dysfunction initiates a tertiary cascade of intermediate signal amplification, predominantly driven by oxidative stress and inflammation. Within this tier, NAD(P)H oxidases, particularly NOX2 and NOX4, generate localized reactive oxygen species (ROS) [15], which concomitantly promote the activation of the NLRP3 inflammasome (comprising NLRP3, ASC, and procaspase-1) and the subsequent maturation of pro-inflammatory cytokines, including interleukin-1β and interleukin-18 [16,17]. This deleterious amplification is further exacerbated when compensatory endogenous antioxidant defenses—mediated by the redox-sensitive transcription factor Nrf2 and its cytoprotective target genes (HO-1, NQO1, and SOD)—are overwhelmed or impaired [18,19,20,21]. Ultimately, these converging intermediate pathways culminate in a terminal tier of phenotypic execution, manifesting as widespread endothelial dysfunction, cellular apoptosis, and maladaptive fibrotic remodelling, which constitute the hallmark tissue architecture of CRS [22,23,24,25]. The gut–kidney–heart axis represents a crucial integrated system in CRS pathogenesis. Specifically, gut dysbiosis leads to an increased intestinal permeability (leaky gut), allowing the translocation of microbiota-derived uremic toxins (such as p-cresyl sulfate and TMAO) into the systemic circulation [26,27]. These toxins act as upstream triggers that exacerbate oxidative stress and mitochondrial dysfunction. Polyphenol supplementation modulates this axis through a stepwise mechanism: first, by restoring microbial diversity and strengthening the intestinal barrier; second, by reducing the systemic load of pro-oxidant toxins [28,29,30]; and finally, by preventing the downstream activation of inflammatory and fibrotic pathways in both cardiac and renal tissues [31,32]. Given this complex pathogenic cascade, increasing attention has been directed toward dietary bioactive compounds capable of simultaneously modulating multiple interconnected pathways across these distinct mechanistic tiers. Polyphenols, a ubiquitous family of plant-derived molecules characterized by aromatic rings bearing one or more hydroxyl groups, are widely distributed in fruits, vegetables, cereals, tea, wine, and medicinal plants [33,34,35,36]. Rather than adopting a strictly chemical classification, the present review focuses on polyphenolic compounds selected for their documented ability to target these interlinked, multi-tiered molecular drivers, thereby providing a robust mechanistic rationale for their investigation as pleiotropic modulators of the cardio-renal continuum (Figure 1).

## 2. Materials and Methods

This narrative review aimed to explore the role of polyphenols in modulating molecular mechanisms involved in cardiorenal syndromes. A comprehensive literature search was performed using PubMed and Google Scholar, covering all articles published between January 2005 and December 2025. The search strategy included combinations of keywords such as “polyphenols”, “cardiorenal syndrome”, “chronic kidney disease”, “cardiovascular disease”, “oxidative stress”, “inflammasome”, and “fibrosis”. Additional references were identified by reviewing the bibliographies of relevant articles and previous reviews. Studies were included if they were original peer-reviewed research articles—encompassing in vivo animal models, in vitro cell cultures, and human clinical studies—published in English within the specified timeframe. Furthermore, eligible studies had to investigate the effects of the selected polyphenols on cardiac and renal outcomes while providing clear mechanistic insights into the molecular drivers of CRS. Conversely, articles were excluded if they were published in languages other than English, or if they consisted of editorials, commentaries, letters to the editor, conference abstracts without full-text availability, or non-peer-reviewed preprints. Finally, studies focusing solely on broad cardiovascular or renal outcomes without isolating the specific molecular or mechanistic data of the targeted polyphenols were also excluded from this review. The selection of polyphenolic compounds for inclusion in this review was mechanistically driven, rather than exhaustive. Compounds were chosen based on preclinical or clinical evidence demonstrating their ability to modulate at least two of the core molecular pathways implicated in cardiorenal dysfunction, namely oxidative stress, inflammasome activation, endothelial dysfunction, and fibrotic remodeling. This rationale allowed us to focus on molecules with multi-target potential, which is particularly relevant for diseases such as CRS where overlapping pathological mechanisms coexist. Specifically, the review highlights bergamot, curcumin, quercetin, catechins, and resveratrol, as these compounds have been most consistently studied in preclinical models of cardiorenal disease and show evidence of mechanistic actions across multiple signaling pathways, including Nrf2/HO-1, NF-κB, GSK-3β/β-catenin, and SIRT1/AMPK signaling. By emphasizing these molecules, the review aims to provide a cohesive mechanistic narrative linking dietary polyphenols to cardiorenal protection. Studies were included if they investigated the effects of these compounds on cardiac and renal outcomes in animal models, cell cultures, or human studies, and provided mechanistic insights relevant to the molecular drivers of CRS. Articles focusing solely on unrelated cardiovascular or renal outcomes without mechanistic data were excluded.

## 3. Polyphenols

Polyphenols are molecules composed of one or more hydroxyl groups (–OH) attached to aromatic phenols, found in vegetables, fruits, legumes, cereals, tea, but also in fish, crustaceans, and algae [37]. While phenolic-like bioactives are occasionally detected in higher marine taxa, such as teleost fish and crustaceans, these organisms lack the enzymatic machinery for the de novo synthesis of classical plant polyphenols; consequently, their presence in marine fauna strictly reflects the bioaccumulation of dietary phytomolecules or their metabolic derivatives rather than endogenous production [38]. Depending on their chemical structure, they can be divided into phenolic acids, which are non-flavonoid polyphenols, further divided into benzoic acid and cinnamic acid, flavonoids, which can be further divided into sub-groups such as anthocyanins, flavanols, flavones, flavanones, polyphenolic amides (including capsaicinoids) [33], stilbenes (of which resveratrol is the most studied), phenolic alcohols, and lignans [39,40,41] (Table 1).

Dietary polyphenols have been studied for their preventive role in cardiovascular, neurodegenerative, and cancer diseases, as well as their glucose-lowering effect [42,43,44,45], demonstrating antioxidant activity in vitro and in vivo, inhibiting ACE activity [46], and modulating signaling cascades such as phosphoinositide 3-kinase (PI3-kinase), Akt/protein kinase B (Akt/PKB), tyrosine kinases, protein kinase C (PKC), and mitogen-activated protein kinase (MAP kinase) [47,48]. Preclinical evidence from in vivo and in vitro models suggests that polyphenolic compounds exert significant nephroprotective effects [8,31]. An emerging and critical mechanism linking renal decline to cardiovascular mortality in CRS is the Fibroblast Growth Factor 23 (FGF23)/Klotho axis. Klotho, an anti-aging protein predominantly expressed in the kidneys, serves as an obligate co-receptor for FGF23, a bone-derived hormone regulating phosphate homeostasis [49,50]. In the early stages of CKD, renal Klotho expression precipitously declines, prompting a compensatory, yet maladaptive, exponential rise in circulating FGF23 levels. This state of Klotho deficiency and FGF23 excess directly contributes to left ventricular hypertrophy (LVH), endothelial dysfunction, and myocardial fibrosis, establishing this signaling pathway as a fundamental “cardiorenal connector” [51,52].

Recent preclinical evidence highlights the capacity of dietary polyphenols to modulate this pivotal pathway, primarily by preventing renal Klotho downregulation. Resveratrol, through its robust activation of the SIRT1 axis, has been shown to upregulate renal Klotho expression, thereby helping to restore the physiological FGF23/Klotho balance and mitigating FGF23-induced cardiac remodelling [53]. Similarly, multi-target polyphenols like curcumin and quercetin have demonstrated the ability to preserve Klotho levels in uremic models [54]. They achieve this by suppressing systemic oxidative stress and NF-κB-mediated inflammation, which are known transcriptional repressors of the *Klotho* gene. Furthermore, by ameliorating epigenetic silencing—such as preventing DNA hypermethylation of the Klotho promoter—and reducing oxidative stress-induced cellular senescence, these bioactives indirectly blunt the cardiotoxic effects of elevated FGF23 [55]. Consequently, the preservation of the Klotho/FGF23 axis provides a novel and robust mechanistic explanation for the cardioprotective efficacy of polyphenols in the setting of progressive renal impairment.

A meta-analysis by Bach et al. [56] evaluated the impact of healthy dietary patterns on the incidence of CKD. The results indicated that adherence to the Mediterranean diet was associated with a significant reduction in CKD risk, while the effects of the DASH diet were more heterogeneous. This evidence supports the concept that polyphenol-rich dietary patterns contribute to renal protection and reinforce the translational relevance of polyphenols for cardiorenal health. Several studies have investigated the therapeutic potential of polyphenols in humans. A systematic review of 28 RCTs (*n* = 20–108) [57] showed that in metabolic syndrome, bergamot extract (6 months) reduced LDL by 22% and triglycerides by 23% (*p* < 0.01). Grape powder (60 g/day for 4 weeks) lowered triglycerides and improved HDL function, while freeze-dried blueberry (45 g/day for 6 weeks) improved endothelial function (*p* < 0.05). Polyphenols from grapes also reduced blood pressure, and glycemic improvements were limited to polyphenol-rich diets and high-dose resveratrol. In CKD, fruit- and vegetable-based diets (up to 5 years) slowed eGFR decline and increased plasma bicarbonate to levels comparable to sodium bicarbonate. Long-term interventions (5 years) reported zero cardiovascular events compared to six in controls (*p* < 0.01). Isolated supplements (e.g., cranberry, resveratrol) showed minimal effects on renal function, and no serious adverse events were reported. Overall, polyphenols exert antioxidant, anti-inflammatory, and endothelial-protective effects, modulating multiple molecular pathways relevant to the cardiorenal axis. Emerging nanoformulations have further improved bioavailability and therapeutic efficacy, supporting their potential as adjunctive interventions in CKD and cardiovascular disease [58].

### 3.1. Bergamot

Bergamot (*Citrus bergamia*), a citrus fruit endemic to Southern Italy, is characterized by a distinctive polyphenolic profile enriched in flavonoids and flavonoid glycosides, including neoeriocitrin, neohesperidin, naringin, rutin, neodesmin, rhoifolin, and poncirin [57,59]. It is crucial to distinguish between different bergamot-derived formulations, as the phytochemical profile varies significantly depending on the source material. Specifically, the Bergamot Leaf Polyphenol Fraction (BLPF) and the Bergamot Fruit Polyphenol Fraction (BFPF) exhibit distinct compositions. Analytical data indicate that the total polyphenol content is approximately 38% higher in the leaves compared to the fruit. While BFPF is characterized by a higher percentage of non-polyphenolic phenols (4.5% vs. 1.8% in BLPF), BLPF shows a superior concentration of total polyphenols (95.5% vs. 79.8%). Furthermore, the internal ratio of flavonoids differs: BLPF contains a higher proportion of flavones (33.1% vs. 21.6% in BFPF), whereas both fractions remain rich in flavanones [60]. These divergent analytical profiles suggest that leaf-derived extracts may offer a more concentrated source of specific bioactives, potentially influencing their comparative antioxidant and anti-inflammatory efficacy in cardiorenal models. These compounds exert pleiotropic antioxidant, anti-inflammatory and metabolic effects, making bergamot a promising candidate for the modulation of cardiorenal syndromes. In a renovascular hypertension model induced by unilateral renal artery ligation combined with DOCA-salt administration, Carresi et al. demonstrated that treatment with bergamot polyphenolic fraction (BPF) significantly reduced mean arterial pressure compared with untreated hypertensive rats. Although normotension was not fully restored, BPF reduced contralateral kidney hypertrophy and volume, improved myocardial strain parameters, and limited early inflammatory and structural reno-cardiac damage, supporting a protective role of bergamot during the initial phases of disease progression [61]. Bergamot has also been investigated in experimental models of cardiorenal metabolic syndrome. In rats fed a high sugar–fat diet, bergamot leaf extract ameliorated insulin resistance, dyslipidemia and systolic blood pressure, leading to attenuation of the metabolic cardiorenal phenotype [62]. Mechanistically, bergamot polyphenols were shown to suppress hepatic lipogenesis, stimulate autophagy and enhance short-chain fatty acid production, thereby linking metabolic regulation with cardiorenal protection [63]. Beyond metabolic effects, BPF modulates key molecular pathways involved in oxidative stress and inflammation. In animal models characterized by heightened reno-cardiac oxidative stress, BPF reduced reactive oxygen species generation, downregulated pro-inflammatory cytokines and prevented apoptotic signaling in renal and myocardial tissues [64]. Complementary in vitro studies demonstrated that bergamot flavonoids preserve endothelial integrity under oxidative stress conditions, improving nitric oxide bioavailability and endothelial-dependent vasodilation [65]. Furthermore, cardioprotective effects of BPF have been reported in doxorubicin-induced cardiomyopathy, where bergamot supplementation attenuated cardiomyocyte injury and fibrotic remodeling, suggesting that its renoprotective properties are paralleled by a direct myocardial benefit [64].

### 3.2. Curcumin

Curcumin, a hydrophobic polyphenol derived from Curcuma longa, is recognized for its antioxidant, anti-inflammatory, and antifibrotic properties, although its clinical application is limited by poor bioavailability and chemical instability [66]. Curcumin induces heme oxygenase-1 (HO-1) in renal tubular cells, an enzyme catalyzing the degradation of heme into carbon monoxide, biliverdin, and free iron, which collectively exert anti-inflammatory and cytoprotective effects in kidney, liver, and lung tissues [67]. In anti-Thy1 glomerulonephritis rat models, intraperitoneal curcumin administration decreased plasminogen activator inhibitor-1 (PAI-1) and transforming growth factor-β (TGF-β), ameliorating proteinuria, and demonstrating an HO-1-dependent antifibrotic effect [68]. In 5/6 nephrectomy (Nx) rat models of chronic kidney disease (CKD) featuring proteinuria, hypertension, and left ventricular hypertrophy, curcumin significantly attenuated cardiac hypertrophy and reduced proteinuria, blood urea nitrogen, and creatinine levels, while modulating GSK-3β, β-catenin, and the calcineurin–NFAT signaling pathway [69,70,71,72]. These molecular effects contribute to the mitigation of cardiac remodeling secondary to CKD. Curcumin also reduces oxidative stress, inflammation, and apoptosis in both renal and cardiac tissues, thereby preserving organ function [73]. To overcome bioavailability limitations, novel curcumin analogues and formulations have been developed. C66, a monocarbonyl analogue, exhibits improved stability and reduces diabetes-associated aortic and renal inflammation, oxidative stress, fibrosis, and apoptosis by suppressing JNK2 and NF-κB pathways [74,75,76]. Theracurcumin, a formulation with enhanced solubility and bioavailability, has been shown to reduce cardiac fibrosis and improve diastolic function in subtotal nephrectomy (SNx) rat models. Theracurcumin treatment attenuated left ventricular hypertrophy, interstitial fibrosis, and myocyte hypertrophy while reducing NLRP3 inflammasome activation and IL-1β secretion in the heart, highlighting its potential in mitigating CKD-associated cardiac inflammation [77,78]. Recent evidence further supports curcumin’s role in cardiorenal syndromes, showing preservation of mitochondrial function, attenuation of ROS, and protection against myocardial and renal injury in ischemia–reperfusion and CKD models [79,80]. Additionally, curcumin modulates fibrotic regulatory axes, such as circ_0008925/miR-204-5p/IL6ST, improving renal fibrosis and lipid metabolism by lowering serum triglycerides, cholesterol, free fatty acids, and LDL levels [81,82]. Clinical studies in CKD patients demonstrate reductions in inflammatory and oxidative markers with curcumin supplementation, although improvements in renal function are modest [83]. This profound discrepancy between robust preclinical antifibrotic efficacy and the failure to achieve hard renal endpoints in human trials stems from several interconnected translational barriers. Primarily, the notoriously poor aqueous solubility, rapid systemic metabolism, and rapid elimination of native curcumin severely restrict its absolute bioavailability, preventing the attainment of therapeutic tissue concentrations despite high oral doses [66,84]. Furthermore, while animal models typically employ short-term, high-dose interventions initiated concurrently with acute disease induction, human clinical trials often feature limited treatment durations applied to patients with long-standing, irreversible fibrotic remodeling. This temporal mismatch is inherently compounded by significant patient heterogeneity; the complex clinical comorbidities, concurrent standard-of-care pharmacological regimens, and diverse etiologies of CKD in human populations critically dilute the targeted molecular effects readily observed in highly controlled, genetically homogeneous experimental models [85,86]. Collectively, these findings support curcumin and its advanced formulations as promising multi-target agents for cardiorenal protection, provided that future clinical trial designs rigorously address these pharmacokinetic and demographic limitations.

Collectively, these findings support curcumin and its advanced formulations as promising multi-target agents for cardiorenal protection.

### 3.3. Quercetin

Quercetin (3,3′,4′,5,7-pentahydroxyflavone) is a naturally occurring polyphenolic flavonoid abundant in vegetables, tea, wine, and fruits, recognized for its anti-inflammatory, antioxidant, and cardioprotective properties [87]. It modulates multiple inflammatory pathways by inhibiting cyclooxygenase (COX), lipoxygenase (LOX), and NF-κB activity, while enhancing antioxidant defenses via the Nrf2 pathway. In vascular endothelial cells, quercetin improves vasodilatory function, reduces reactive oxygen species (ROS) production, inhibits NOX2 activation, and lowers pro-inflammatory cytokines such as IL-1β, TNF-α, and IL-6, contributing to blood pressure reduction and endothelial homeostasis [88]. In the kidney, quercetin has been shown to exert protective effects in experimental models of renal injury. It inhibits NF-κB activity, attenuates TGF-β1 signaling, and reduces extracellular matrix deposition, in part through modulation of macrophage polarization by suppressing pro-inflammatory M1 and promoting reparative M2 phenotypes [89]. Quercetin also inhibits ferroptosis, an iron-dependent form of regulated necrosis, in renal tubular epithelial cells in diabetic kidney disease (DKD), reducing intracellular iron and malondialdehyde (MDA) levels, increasing glutathione, and limiting ROS accumulation, thereby slowing disease progression [90,91]. Mechanistically, quercetin influences lipid metabolism and energy homeostasis in DKD via the PPARA/PPARG-UCP1 axis, decreasing lipid accumulation, stabilizing glucose levels, and improving insulin sensitivity [92]. In hypertensive 2K1C rat models, quercetin reduced TGF-β levels and coronary perfusion pressure, although it did not significantly alter left ventricular structure, blood pressure, or MMP activity [93]. Beyond these effects, recent studies have revealed additional cardio-renal mechanisms: quercetin mitigates renal fibrosis through mitophagy induction via the SIRT1/PINK1/Parkin pathway, reducing tubular cell senescence [94]. It also suppresses ferroptosis in CKD models by downregulating the EGFR/ACSL4 axis, thereby decreasing iron accumulation and oxidative damage [95]. Quercetin inhibits epithelial–mesenchymal transition (EMT) and matrix deposition by suppressing Hedgehog signaling and transcription factors such as Snail, Slug, ZEB-1, and Twist, limiting renal fibrogenesis [96]. Furthermore, quercetin modulates the TGF-β/miR-21 axis, enhancing PTEN and TIMP3 expression, which reduces extracellular matrix accumulation in tubular cells [97]. It also attenuates inflammatory and profibrotic signaling through the SMAD pathway, decreasing TNF-α, TGF-β1, MDA, and SMAD2/3/4 expression while upregulating SMAD7, further supporting anti-fibrotic effects [98]. Collectively, these findings indicate that quercetin exerts multifaceted cardio-renal protective effects by modulating inflammation, oxidative stress, fibrosis, lipid metabolism, and cell death pathways, highlighting its therapeutic potential in chronic kidney disease and associated cardiovascular complications. These findings are supported by a recent systematic review and meta-analysis that showed how quercetin supplementation significantly reduces serum uric acid, creatinine, fractional excretion of uric acid and blood urea nitrogen (BUN) levels, improving oxidative stress and inflammatory markers, including malondialdehyde (MDA), superoxide dismutase (SOD), interleukin-6 (IL-6), and tumor necrosis factor-α (TNF-a). Quercetin also reduces lipid parameters such as triglycerides (TG) and very-low-density lipoprotein (VLDL) (*p* < 0.05) [99].

### 3.4. Catechins

Catechins are a group of flavonoids widely present in dietary sources including apples, blueberries, gooseberries, grape seeds, kiwi, strawberries, green and black tea, red wine, beer, cacao liquor, chocolate, and cocoa [100]. The principal catechins in green tea, such as (−)-epigallocatechin-3-gallate (EGCG), epicatechin, epigallocatechin, epicatechin gallate, and catechin itself, exhibit robust antioxidant properties and modulate multiple molecular pathways involved in cardiovascular and renal health [101,102]. Numerous preclinical studies demonstrate that catechins exert both cardio- and reno-protective effects. EGCG has been shown to attenuate myocardial remodeling and improve cardiac energy metabolism in pressure overload models, thereby reducing fibrosis, hypertrophy markers such as B-type natriuretic peptide, and ameliorating systolic dysfunction [103]. In renal contexts, EGCG mitigates diabetic kidney disease (DKD) by suppressing pro-inflammatory signaling pathways including TXNIP/NLRP3/IL-1β, which are central to inflammasome activation and subsequent inflammatory damage in podocytes and kidney parenchyma [104]. Other studies in diabetic models report that EGCG reduces urinary protein excretion, oxidative stress markers, angiotensin II levels, and expression of NADPH oxidase subunits, ultimately preserving renal architecture and function [105]. EGCG also attenuates salt-induced hypertension and renal injury in salt-sensitive models, lowering blood pressure, reducing proteinuria, and decreasing creatinine clearance, effects associated with diminished oxidative stress, macrophage infiltration and inflammatory signaling in renal tissues [106]. In obstructive nephropathy models, EGCG reduces oxidative stress and inflammation, suggesting its broader renoprotective potential across distinct kidney injury pathways [107]. Beyond direct organ-specific effects, catechins improve endothelial function and vascular reactivity by modulating vasodilatory and vasoconstrictive mediators, reducing lipid peroxidation, and inhibiting smooth muscle cell proliferation, which are key processes in cardiorenal disease progression [108]. Mechanistically, catechins activate the HO-1 and Nrf2 pathways, enhancing endogenous antioxidant defenses and countering ROS-driven damage in both renal and cardiac cells [109]. EGCG prevents epithelial–mesenchymal transition (EMT) in renal tubular cells via Nrf2-dependent mechanisms, thereby limiting fibrotic remodeling [110]. Additionally, catechins demonstrate the ability to attenuate ER stress and downregulate NLRP3 inflammasome components in tubular injury models, further contributing to reduced inflammation and fibrosis [111]. Collectively, these studies highlight catechins, particularly EGCG, as multi-target modulators of oxidative stress, inflammation, fibrosis, and energy metabolism, with significant implications for the prevention and mitigation of chronic kidney disease and cardiovascular dysfunction. However, it is crucial to note that the biological effects of catechins, particularly EGCG, are highly dose-dependent, often exhibiting a biphasic or hormetic response. While low to moderate dietary concentrations confer robust antioxidant and anti-inflammatory benefits, toxicological studies have reported that very high, pharmacological doses of EGCG can paradoxically exert pro-oxidant effects. At elevated concentrations, EGCG can undergo auto-oxidation, leading to the excessive generation of reactive oxygen species (ROS) such as hydrogen peroxide. This pro-oxidant shift can induce cellular toxicity, exacerbate endoplasmic reticulum stress, and lead to hepatotoxicity or further tissue damage [112,113]. Therefore, translating these preclinical findings into clinical applications requires careful optimization of dosing to maximize cardiorenal protection while avoiding concentration-dependent adverse effects. Collectively, these studies highlight catechins, particularly EGCG, as multi-target modulators of oxidative stress, inflammation, fibrosis, and energy metabolism, with significant implications for the prevention and mitigation of chronic kidney disease and cardiovascular dysfunction.

### 3.5. Resveratrol

Resveratrol (RSV, 3,4′,5-trihydroxy-trans-stilbene) is a polyphenolic stilbene derivative predominantly found in red wine and grapes. It has been extensively studied for its antioxidant, anti-inflammatory, and endothelial-protective properties. RSV enhances nitric oxide bioavailability and improves vascular function in both in vitro and in vivo models [114,115]. In diabetic kidney disease, RSV exerts renoprotective effects by reducing oxidative stress, attenuating pro-inflammatory cytokines, and improving endothelial function [116,117]. In models of ischemia–reperfusion injury, RSV decreased serum creatinine and BUN through inhibition of nitric oxide synthase, while sustained RSV infusion in rats promoted renal vasodilation and increased natriuresis without altering GFR [118,119]. RSV prevents the decline of endogenous antioxidants such as superoxide dismutase (SOD) and glutathione reductase, mitigating cardiac dysfunction in models of diabetes and renal hypertension [120,121]. It restores endothelial responsiveness to acetylcholine and exhibits sympatholytic activity by preventing heart rate elevation in type 2 diabetic and hypertensive rats [122,123]. Beyond its hemodynamic effects, RSV induces the Nrf2/ARE pathway; in spontaneously hypertensive rats, RSV restores Nrf2 activity, upregulating endogenous antioxidant enzymes such as SOD and HO-1, thereby reducing systemic hypertension and renal inflammation [124]. Sirtuins are a family of NAD+-dependent deacetylases involved in energy metabolism, genomic stability, and stress resistance [125,126]. RSV directly activates SIRT1, modulating multiple molecular pathways including AMPK, Nrf2, and NF-κB [127]. In CKD-induced left ventricular remodeling, RSV upregulates MnSOD in cardiomyocytes, reducing oxidative stress. Loss of a SIRT1 allele exacerbates cardiorenal pathology in 5/6 nephrectomy models, indicating that RSV’s effects are at least partially SIRT1-dependent. SIRT1 deacetylates FoxO1, a transcription factor involved in cardiac aging, insulin signaling, and renal protection [128,129]. In the 2K1C hypertension model, RSV reduced systolic blood pressure, whole heart hypertrophy, and ventricular collagen deposition more effectively than captopril [130]. However, this effect has been observed exclusively in animal models and it does not indicate a clinical superiority of resveratrol. In acute kidney injury induced by isoproterenol, liposomal RSV mitigated renal damage via downregulation of MAPK, cystatin C, Fas, and lipocalin-2 [131]. Additional studies have highlighted RSV’s role in preserving cardiac function, preventing arrhythmias, and modulating mitochondrial dynamics [132]. RSV also influences the gut–kidney–heart axis by modulating gut microbiota composition and reducing uremic toxins such as indoxyl sulfate and TMAO, which are implicated in CKD-associated cardiovascular damage [133]. Beyond cardiorenal protection, RSV may contribute to healthy aging and neuroprotection. SIRT1 activation by RSV promotes deacetylation of FoxO1, enhancing insulin sensitivity and glucose uptake in skeletal muscle, which is relevant in elderly and diabetic populations [134]. Long-term RSV supplementation in aging mice reduced age-related cardiac fibrosis, improved ventricular compliance, and mitigated oxidative stress [135]. Furthermore, moderate wine consumption, which provides RSV and other polyphenols, has been associated with cardiovascular and renal benefits, improved endothelial function, and potentially slower progression of chronic renal diseases [136,137]. Clinical evidence, while limited by small sample sizes and variability in dosage, suggests that RSV improves flow-mediated dilation, insulin sensitivity, postprandial glucose control, and oxidative stress markers in humans with metabolic syndrome or diabetes [138]. Novel formulations such as liposomal RSV are being developed to overcome poor bioavailability and ensure higher therapeutic efficacy [131,139]. The main preclinical studies investigating the effects of polyphenolic compounds in cardiorenal syndrome models, including their respective experimental designs and key molecular outcomes, are summarized in Table 2.

## 4. Discussion

Cardiorenal syndrome represents a complex interplay between cardiac and renal dysfunction, where oxidative stress, inflammation, and mitochondrial derangements are central pathogenic mechanisms. While several studies provide a mechanistically sound hypothesis for the attenuation of cardiorenal connectors—such as oxidative stress, fibrosis, and neurohormonal overactivation—clinical translation is still in its early stages. The preclinical evidence reviewed here indicates that polyphenols—including bergamot, curcumin, quercetin, catechins, and resveratrol—simultaneously modulate multiple molecular pathways involved in CRS, providing a mechanistic rationale for their potential cardioprotective and renoprotective effects. A recurring theme across multiple studies is the ability of polyphenols to regulate cellular redox balance. Bergamot polyphenolic fraction (BPF) reduces reactive oxygen species (ROS) and inflammatory cytokines in renal and cardiac tissues while preserving endothelial function and vasodilation [59,61,65]. Curcumin and its analogs (C66, Theracurcumin) attenuate oxidative stress in CKD and diabetic nephropathy models through induction of heme oxygenase-1 (HO-1) and inhibition of NADPH oxidase activity, contributing to reduced cardiac hypertrophy and renal fibrosis [67,78,90]. Quercetin protects renal tubular cells from ferroptosis by decreasing ROS and malondialdehyde (MDA) levels while enhancing glutathione and activating Nrf2-dependent antioxidant pathways [91,95,98]. Catechins, particularly epigallocatechin-3-gallate (EGCG), exert antioxidant effects via upregulation of Nrf2/ARE-driven genes and inhibition of NADPH oxidase, improving both cardiac and renal tissue redox status [140]. Resveratrol exerts similar antioxidative effects, enhancing SOD activity, restoring endothelial NO bioavailability, and mitigating oxidative stress–induced cardiac and renal injury [123,124]. Furthermore, resveratrol interacts with SIRT1 and the FoxO1 axis, which reduces cardiomyocyte apoptosis, promotes mitochondrial homeostasis, and improves cardiac remodeling in CKD models [69,141].

Collectively, these studies suggest that polyphenols converge on redox-sensitive pathways—including Nrf2, HO-1, NADPH oxidase, and SIRT1/FoxO1—to counteract oxidative stress, a pivotal driver of CRS pathophysiology. Chronic inflammation contributes to structural and functional deterioration in CRS, and polyphenols effectively attenuate pro-inflammatory signaling and immune cell infiltration. Bergamot flavonoids reduce NF-κB activation and lower TNF-α, IL-1β, and IL-6 levels in cardiac and renal tissues, limiting fibrosis and cellular injury [61,65]. Curcumin and C66 analogs inhibit NF-κB and MAPK pathways, reducing the expression of ICAM-1, VCAM-1, and MCP-1 in renal epithelial cells [74,142]. Quercetin modulates macrophage polarization, suppressing M1-mediated inflammatory responses while promoting M2-associated reparative activity in the kidney [89]. Catechins and EGCG reduce endothelin-1–mediated NADPH oxidase activation, thereby indirectly lowering vascular inflammation [143]. Resveratrol suppresses pro-inflammatory cytokine production, macrophage infiltration, and NLRP3 inflammasome activation in both cardiac and renal tissues, contributing to attenuation of fibrosis and functional decline [144,145]. This anti-inflammatory action is further amplified by the emerging role of the “gut-kidney-heart” axis; polyphenols modulate the microbiota, reducing the translocation of gut-derived uremic toxins like TMAO and indoxyl sulfate, which are known to fuel systemic chronic inflammation and exacerbate cardiovascular damage [146].

Endothelial dysfunction is a hallmark of CRS and contributes to hypertension and vascular remodeling. Polyphenols improve endothelial function through multiple mechanisms, including enhanced NO bioavailability, Ca^2+^-dependent vasodilation, and inhibition of vasoconstrictive mediators such as endothelin-1. Bergamot, quercetin, and catechins have all been shown to restore endothelial-dependent vasodilation in animal models of hypertension and CKD [59,65,143]. Resveratrol enhances endothelial function by promoting eNOS activity and activating SIRT1, which improves vascular relaxation and reduces arterial stiffness [147,148]. Moreover, moderate consumption of red wine, a natural source of resveratrol and other polyphenols, has been associated with improved endothelial function and reduced oxidative stress, suggesting translational relevance for CRS patients [136,147].

**Figure 2:**
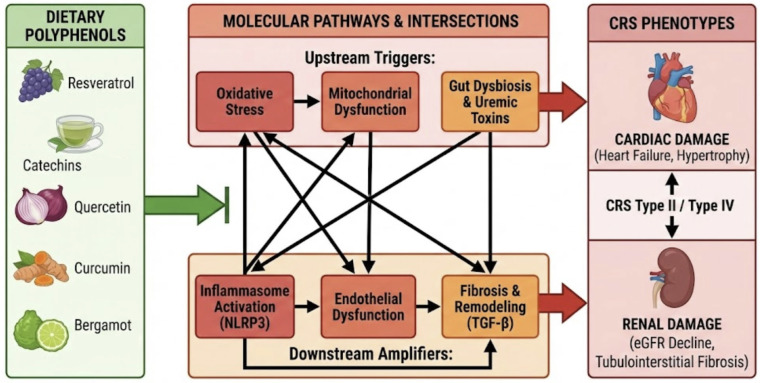
Dietary polyphenols: molecular pathways and CRS phenotypes.

Mitochondrial dysfunction is increasingly recognized as a critical mediator of CRS progression, where polyphenols preserve mitochondrial integrity by modulating bioenergetics and reducing ROS production. Resveratrol activates SIRT1 and AMPK, promoting mitochondrial biogenesis and improving ATP production in cardiomyocytes and renal tubular cells [11,134]. Curcumin and catechins exert similar effects by enhancing autophagy and mitophagy, particularly via the PINK1/Parkin-mediated pathways, which prevent the accumulation of damaged organelles and reduce pro-apoptotic signaling [94,149]. This “cellular cleaning” or proteostasis is vital for maintaining cellular health in both the heart and kidney, effectively linking metabolic regulation to the suppression of the NLRP3 inflammasome and preventing the progression toward end-stage organ failure [17]. In models of metabolic CRS, bergamot polyphenols improved insulin sensitivity, reduced dyslipidemia, and decreased hepatic lipogenesis, linking metabolic regulation to renal and cardiac protection [59]. Molecular pathways and CRS phenotypes are illustrated in Figure 2.

Despite this compelling mechanistic framework, the translation of polyphenols into clinical practice for CRS is fraught with challenges, and the broader literature includes several neutral or negative findings. A primary limitation of the current evidence base is the over-reliance on in vitro and animal models utilizing supraphysiological doses that are largely unattainable in humans. In clinical settings, polyphenols suffer from notably poor systemic bioavailability, rapid phase II metabolism, and swift elimination. Consequently, several randomized controlled trials have yielded neutral results; for instance, some clinical studies investigating isolated, high-dose supplementation of resveratrol or quercetin in patients with established cardiovascular disease or advanced CKD have failed to demonstrate significant improvements in primary endpoints, such as estimated glomerular filtration rate (eGFR) trajectories or left ventricular ejection fraction.

Furthermore, potential negative or adverse effects cannot be overlooked. As noted with high-concentration catechins, supraphysiological doses of polyphenolic extracts can induce a pro-oxidant shift, potentially causing mild gastrointestinal distress, interfering with cytochrome P450 drug metabolism—a critical concern for polypharmacy patients with CRS—or paradoxically exacerbating cellular stress. Another significant limitation is the extensive heterogeneity among human trials, driven by varying study designs, lack of standardized formulations, and profound inter-individual differences in gut microbiota, which dictates the generation of active circulating metabolites. While novel formulations, including nanocapsules, SNEDDS, and analogs with improved pharmacokinetic properties (e.g., Theracurcumin, C66, liposomal resveratrol), show promise in enhancing efficacy while minimizing side effects [139,150,151], current evidence still lacks the large-scale, rigorously controlled trials needed to confirm their clinical utility. The low bioavailability of parent polyphenol compounds is further complicated by their extensive transformation by gut microbiota into bioactive metabolites (e.g., urolithins from ellagitannins or phenolic acids from flavonoids). These metabolites often circulate at higher concentrations and for longer durations than the original compounds, suggesting that the clinical efficacy of polyphenols may depend more on individual microbiotic profiles than on the administered dose itself [152,153]. This ‘inter-individual variability’ represents a major hurdle for standardized clinical protocols. Furthermore, epidemiological evidence supports potential benefits of moderate polyphenol-rich diets, such as the Mediterranean diet and controlled red wine consumption, in reducing cardiovascular and renal risk, as well as age-related decline [154,155].

Importantly, polyphenols target multiple intersecting pathways—oxidative stress, inflammation, endothelial dysfunction, and mitochondrial impairment—positioning them as attractive adjunctive therapies in CRS. In conclusion, polyphenols represent multifunctional natural bioactives capable of modulating redox balance, inflammatory responses, endothelial function, and mitochondrial homeostasis in CRS. Future research should focus on standardized long-term clinical trials to evaluate efficacy, optimal dosing, and combinatorial strategies with existing pharmacotherapies.

## 5. Conclusions

In conclusion, cardiorenal syndrome represents a formidable clinical challenge due to the complex bidirectional nature of organ damage, where oxidative stress, chronic inflammation, and mitochondrial impairment act as synergistic drivers of pathological progression. The preclinical evidence synthesized in this review underscores that polyphenols—specifically bergamot, curcumin, quercetin, catechins, and resveratrol—do not merely function as conventional antioxidants but rather as pleiotropic modulators capable of simultaneously intercepting multiple molecular pathways at the crossroads of the heart-kidney axis.

The therapeutic potential of these natural bioactives lies in their “multitarget” approach: enhancing endogenous cytoprotective defenses via the Nrf2/HO-1 axis, suppressing systemic and local “inflammaging” through the inhibition of the NLRP3 inflammasome, and preserving cellular proteostasis via SIRT1/PINK1/Parkin-mediated mitophagy. These mechanisms collectively break the self-perpetuating feedback loop of cardiorenal injury.

While significant hurdles remain—primarily concerning the limited systemic bioavailability and rapid metabolism of these compounds—the emergence of next-generation drug-delivery systems, including targeted nano-formulations and synthetic analogs with optimized pharmacokinetics, provides a promising trajectory for clinical translation. Moving forward, large-scale, standardized clinical trials are essential to establish precise dosing regimens and to evaluate the safety and efficacy of polyphenols as adjunctive therapies alongside standard-of-care pharmacological treatments. Nevertheless, the current body of preclinical data suggests that integrating nature’s bioactives into the cardiorenal potential candidate compounds represents a promising adjunction to drugs recommended by guidelines, such as SGLT2 inhibitors and RAAS blockers. This integration can attenuate organ fibrosis, improve metabolic homeostasis, and ultimately refine the prognosis of patients affected by this multifaceted systemic condition.

## Figures and Tables

**Table 1:** Major dietary polyphenols, their chemical subclasses, common dietary sources, and key mechanisms relevant to cardiorenal syndromes.

Compound	Subclass	Dietary Sources	Cardiorenal Mechanisms
**Quercetin** [19,20,34]	Flavonol	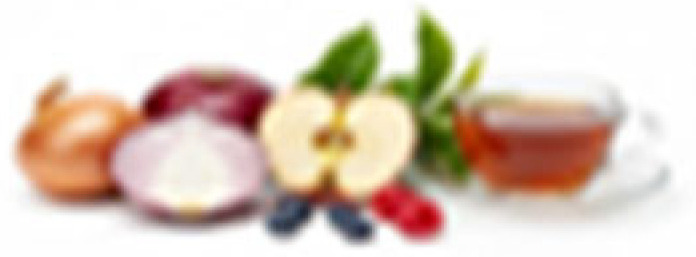	Anti-inflammatory (NF-κB inhibition), antioxidant (Nrf2 activation), inhibits TGF-β-mediated fibrosis, reduces ROS and blood pressure, protects kidney and myocardium in hypertensive/diabetic models
**Catechins (EGCG, epicatechin)** [20,35,36]	Flavanols	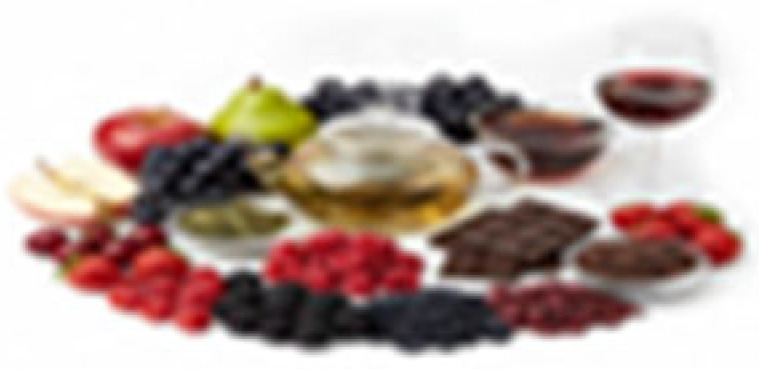	Antioxidant, modulates HO-1 pathway, reduces renal fibrosis and cardiac oxidative stress, improves endothelial function
**Resveratrol** [20,37,38]	Stilbene	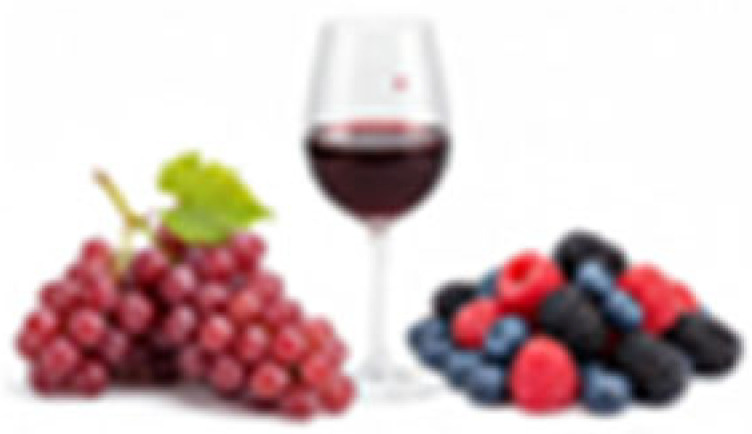	Activates SIRT1/Nrf2, antioxidant, antifibrotic, improves endothelial function, reduces cardiac hypertrophy and renal inflammation
**Curcumin** [28,31]	Polyphenolic diarylheptanoid	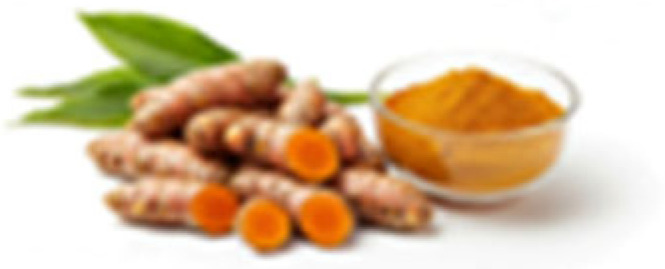	Induces HO-1, antioxidant, antifibrotic (GSK-3β/β-catenin pathway), inhibits NLRP3 inflammasome, improves renal and cardiac remodeling in CKD models
**Bergamot polyphenolic fraction (BPF)** [28]	Flavonoid glycosides	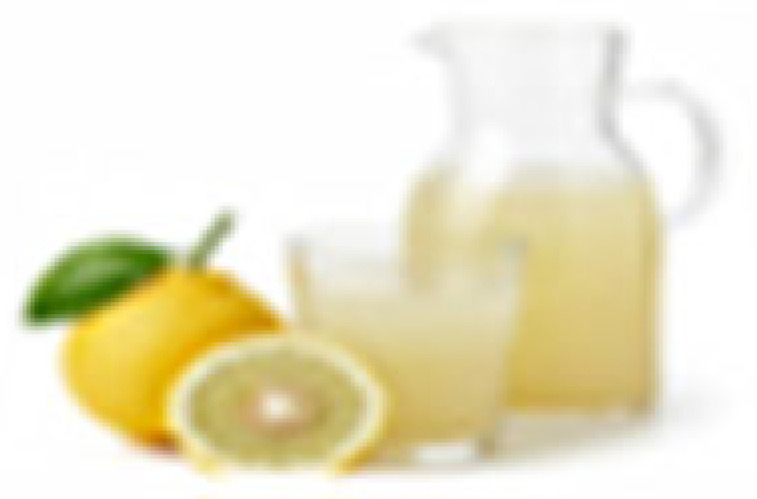	Reduces MAP, improves cardiac strain, protects kidney function, modulates inflammation and oxidative stress in hypertensive rats
**Anthocyanins** [20,27]	Flavonoid	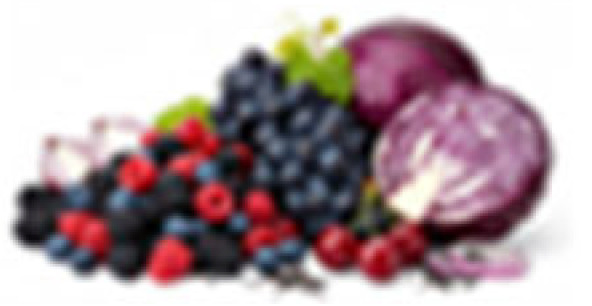	Antioxidant, reduces vascular inflammation, mitigates renal oxidative stress
**Lignans** [21,27]	Non-flavonoid polyphenols	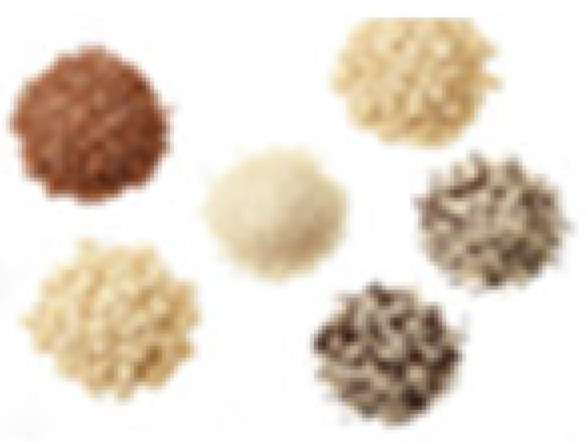	Antioxidant, modulates lipid metabolism, may reduce renal fibrosis
**Phenolic acids (e.g., caffeic, ferulic acid)** [20,21]	Non-flavonoid polyphenols	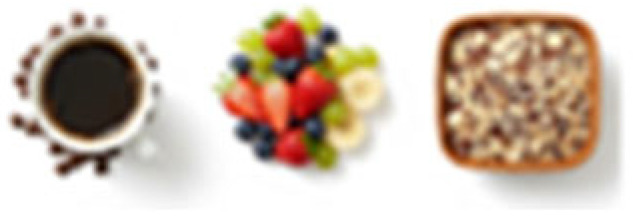	Antioxidant, anti-inflammatory, may attenuate endothelial dysfunction and kidney injury

**Table 2:** Summary of key preclinical studies investigating the therapeutic effects of polyphenols in cardiorenal syndrome (CRS) models.

Compound	Model/Population	Intervention/Dose	Main Outcomes	Mechanism of Action
**Bergamot**	RAL DOCA-Salt rats [61]	Bergamot polyphenolic fraction (BPF)	↓ MAP, ↓ contralateral kidney weight/volume, improved cardiac strain	Antioxidant, anti-inflammatory (↓ NF-κB, ↓ ROS)
	Rats fed high sugar–fat diet [62]	Bergamot leaf extract	Improved insulin resistance, dyslipidemia, ↓ BP, mitigated cardiorenal metabolic syndrome	↓ hepatic lipogenesis, ↑ autophagy, ↑ SCFA production
**Curcumin**	Anti-Thy 1 glomerulonephritis rats [68]	Curcumin 10–200 mg/kg i.p.	↓ PAI-1, ↓ TGF-β, ↓ proteinuria	HO-1 induction, antifibrotic
	Nx rats (CKD) [70]	Curcumin 150 mg·kg^−1^·day^−1^	↓ cardiac hypertrophy, ↓ proteinuria, ↓ BUN/creatinine	↓ GSK-3β phosphorylation, ↓ β-Catenin, ↓ Calcineurin/NFAT
	CDK models [75]	C66 (curcumin analog)	↓ renal inflammation, oxidative stress, fibrosis, apoptosis	JNK2 inhibition, ↓ NF-κB, antioxidant
	SNx rats [77]	Theracurcumin	↓ LV hypertrophy, ↑ chamber compliance, ↓ interstitial fibrosis, ↓ NLRP3, ↓ IL-1β	↓ NLRP3 inflammasome, antifibrotic
**Quercetin**	Mouse kidney injury, cell culture [89]	Quercetin	↓ NF-κB, ↓ TGF-β1, ↓ matrix deposition, modulated M1/M2 macrophages	Anti-inflammatory, antifibrotic
	DKD rats [90]	Quercetin	Inhibited ferroptosis, ↓ ROS, ↑ GSH, ↓ MDA	Antioxidant, ferroptosis inhibition
	DKD rats [91]	Quercetin	↓ lipid accumulation via PPARA/PPARG-UCP1 axis	Lipid metabolism regulation
	2K1C hypertensive rats [93]	Quercetin	↓ TGF-β, ↓ coronary perfusion pressure	Vascular protection
**Catechin**	Diabetic nephropathy [104]	EGCG	↓ renal injury, ↓ TGF-β, ↑ HO-1, renoprotective	Antioxidant, anti-inflammatory
	DOCA-salt rats [8]	Epicatechin	↓ NADPH oxidase, ↓ ET-1, ↑ Nrf2, cardiac and renal protection	Antioxidant, endothelial protection
**Resveratrol**	Diabetic kidney disease [116]	RSV	↓ oxidative stress, ↑ NO, ↓ pro-inflammatory cytokines	Antioxidant, endothelial function
	Ischemia–reperfusion rats [118]	RSV	↓ creatinine, ↓ BUN via NOS inhibition	Antioxidant, renoprotective
	Rats (sustained infusion) [119]	RSV	Renal vasodilation, ↑ natriuresis	Renal hemodynamic modulation
	Diabetic/renal hypertensive rats [120]	RSV	↑ SOD/GSH, improved cardiac function, restored endothelial response	Antioxidant, sympatholytic
	SHR rats [122]	RSV	↑ Nrf2/HO-1, ↓ renal inflammation	Antioxidant, anti-inflammatory
	CKD models [128]	RSV	↑ MnSOD, SIRT1/FoxO1 mediated ↓ oxidative stress, ↓ cardiorenal remodeling	SIRT1 activation, FoxO1 deacetylation
	2K1C hypertensive rats [130]	RSV	↓ SBP, ↓ ventricular hypertrophy & collagen deposition	Anti-hypertrophic, antioxidant
	Isoproterenol-induced AKI [131]	Liposomal RSV	↓ MAPK, ↓ cystatin C, ↓ Fas, ↓ lipocalin-2, renoprotective	Anti-inflammatory, antioxidant
	Aging models/humans [136]	RSV/Moderate red wine	↑ SIRT1, improved cardiac function, glucose metabolism, neuroprotection, ↓ risk dementia	SIRT1 activation, antioxidant, anti-aging

## Data Availability

This article is a review and does not contain any new studies with human participants performed by any of the author.

## Abbreviations

2K1C	two-kidney, one-clip
ACE	angiotensin-converting enzyme
ACSL4	acyl-CoA synthetase long-chain family member 4
AKI	acute kidney injury
Akt	AKT serine/threonine kinase.
AMPK	AMP-activated protein kinase
ATP	adenosin triphosphate
ARE	Antioxidant Response Element
ASC	Apoptosis-associated Speck-like protein
BPF	bergamot polyphenolic fraction
BUN	blood urea nitrogen
C66	(2E,6E)-2,6-bis[(2-trifluoromethyl)benzylidene]cyclohexanone
CKD	chronic kidney disease
COX	cyclooxygenase
CRS	Cardiorenal syndrome
DASH	dietary approaches to stop hypertension
DKD	diabetic kidney disease
DOCA	Deoxycorticosterone Acetate-salt model
EGCG	epigallocatechin-3-gallate
EGFR	epithelial growth factor receptor
EMT	epithelial–mesenchymal transition
ER	endoplasmic reticulum
FoxO1	Forkhead transcription factor
GFR	glomerular filtration rate
GSK-3β/β-catenin	glycogen synthAse kinase
HDL	high-density lipoprotein
HO-1	heme oxygenase-1
ICAM-1	intercellular adhesion molecule 1
IL-1β	interleukin 1 beta
IL-18	interleukin 18
IL-6	interleukin 6
JNK2	c-Jun N-terminal kinase 2
LDL	low-density lipoprotein
LOX	lipoxygenase
MAPK	mitogen-activated protein kinase
MCP-1	monocyte chemoattractant protein-1
MDA	malondialdehyde
miR-21	microRNA
MMP	matrix metalloproteinase
MnSOD	manganese superoxide dismutase
NFAT	nuclear factor of activated T cells
NF-κB	Nuclear Factor kappa-light-chain-enhancer of activated B cells
NLRP3	NOD-, LRR- and pyrin domain-containing protein 3
NO	nitric oxide
NOX	NAD(P)H oxidases
NQO1	NAD(P)H quinone dehydrogenase 1
Nrf2	Nuclear factor erythroid 2–related factor 2
Nx	Nefrectomy
OH	hydroxyl groups
PAI-1	plasminogen activator inhibitor-1
PPARA/PPARG-UCP1	Peroxisome Proliferator-Activated Receptor Alpha/Peroxisome Proliferator-Activated Receptor Gamma uncoupling Protein 1
PI3-kinase	phosphoinositide 3-kinase
PINK1	PTEN-induced kinase 1
PKB	protein kinase B
PTEN	Phosphatase and tensin homoloG
RAAS	renin–angiotensin–aldosterone system
RCT	randomized-controlled trial
ROS	Reactive oxygen species
RSV	Resveratrol
SCFA	short chain fatty acids
SGLT2	sodium-glucose co-transporter 2
SIRT1	sirtuin
SMAD	suppressor of mothers against decapentaplegic
SNEDDS	Self-Nanoemulsifying Drug Delivery System
SNx	subtotal nephrectomy
SOD	superoxide dismutase
TGF-β	transforming growth factor beta
TIMP3	Tissue Inhibitor of Metalloproteinases 3
TMAO	Trimethylamine N-oxide
TNF-α	tumor necrosis factor alpha
TXNIP	Thioxiredoxin-Interacting Protein
VCAM-1	vascular cell adhesion molecule 1
XBP1	X-box binding protein 1
ZEB-1	Zinc Finger E-box-binding Homeobox 1
